# KRAS G12C-Mutant Non-Small-Cell Lung Adenocarcinoma: First Documented Report in the Arabian Gulf

**DOI:** 10.7759/cureus.27090

**Published:** 2022-07-21

**Authors:** Abdullah S Alsulaiman, Siraj B Alharthi, Ahmed S Albariqi, Rasha A Mutabaqani, Fawzi F Bokhari, Islam M Tayeb, Dalia R Alharthi, Muhammad U Tariq, Yasser H Babaier

**Affiliations:** 1 Molecular Diagnostic Unit, Alhada Armed Forces Hospital, Taif, SAU; 2 Biological Sciences, King Abdulaziz University, Jeddah, SAU; 3 Academic Affairs, Directory of Armed Forces Hospitals, Taif, SAU; 4 Physical Therapy Department, Alhada Armed Forces Hospital, Taif, SAU; 5 Histopathology Unit, Alhada Armed Forces Hospital, Taif, SAU; 6 Molecular Virology Unit, Alhada Armed Forces Hospital, Taif, SAU

**Keywords:** arabian gulf, luad, g12c, kras, ras, middle east, saudi arabia, adenocarcinoma, lung cancer

## Abstract

We report the first documented case series of two lung adenocarcinoma patients demonstrating Kirsten rat sarcoma viral oncogene homolog (KRAS) G12C mutations by reverse transcription-polymerase chain reaction techniques from Saudi Arabia. Both patients were males aged 64 and 76 years. The first had a heavy smoking history, while the second did not report any history of smoking. The tumor subtype was identified to be non-mucinous lung adenocarcinoma in both cases. The younger patient presented with generalized lymphadenopathy and a right-sided lung mass lesion, while the older patient exhibited stage III-A left lung adenocarcinoma that required rapid response. An initial examination of the first case showed a right-sided mediastinal shift, bilateral neck lymphadenopathy, and poorly differentiated neoplasm from a right supraclavicular core biopsy, leading to treatment with palliatives along with regular checkups. The second case was afebrile after being confirmed to be vitally stable and laboratory testing (Neutr 100). Further studies, specifically on large numbers of patients from the Arabian Gulf, are needed to confirm significant differences between the national and international populations. Additionally, future studies should investigate more differences in the differentiation of KRAS-mutant lung adenocarcinoma between patients from the Arabian Gulf and others.

## Introduction

Lung cancer is one of the most prevalent types of cancer worldwide, accounting for 1.6 million deaths (or 19% of all cancer deaths) each year [1,2]. Non-small-cell lung cancer (NSCLC) accounts for approximately 85% of all lung cancers [3,4]. Lung adenocarcinoma and lung squamous cell carcinoma are the two most frequent subtypes of NSCLC, accounting for over 85% of all NSCLC cases [5].

Among the most frequently mutated genes in lung adenocarcinoma patients are the oncogenes Kirsten rat sarcoma viral oncogene homolog (KRAS) (32%) and epidermal growth factor receptor (EGFR) (27%), as well as the tumor suppressor genes TP53 (46%), Kelch-like ECH-associated protein 1 (KEAP1) (19%), serine/threonine kinase 11 (STK11) (17%), and neurofibromin 1 (NF1) (11%). On the other hand, the oncogene nuclear factor erythroid-derived 2-like 2 (NFE2L2) (19%) and the tumor suppressor genes TP53 (90%) and cyclin-dependent kinase inhibitor 2A (CDKN2A) (70%) are reported to be the most frequently altered genes in lung squamous cell carcinoma patients [6]. Furthermore, programmed death-ligand 1 (PD-L1)/programmed cell death protein 1 (PD-1) expression has been shown to be aberrant in anywhere from 19% to 100% of NSCLC patients [7-11]. Several of these genes have been linked to various other cancers. Notably, however, RAS gene mutations have been reported to be responsible for 30% of all human cancers, including 90% of pancreatic, lung, and colon cancers. K-RAS, H-RAS, and N-RAS are three forms of RAS proteins that act as molecular switches that are triggered by binding to guanosine triphosphate (GTP), a crucial nucleotide in cell process control [12]. Its mechanism is simple, wherein the protein becomes inactive whenever its GTPase activates, naturally leading to the conversion of GTP to guanosine diphosphate (GDP). Reported RAS carcinogenic mutations, however, cause the loss of internal GTPase activity, resulting in a permanently activated protein [13]. More specifically, however, the KRAS gene, which is the most commonly mutated among the three aforementioned genes, is located on chromosome 12p12.1. The two most common KRAS mutations in NSCLC, namely, G12C (∼40%) and G12V (∼22%), emerge due to G/T transversions [14-16].

We report two cases of lung adenocarcinoma harboring KRAS G12C mutations, which are the first to be reported in the Arabian Gulf according to our literature review.

## Case presentation

Case one

**Figure 1:**
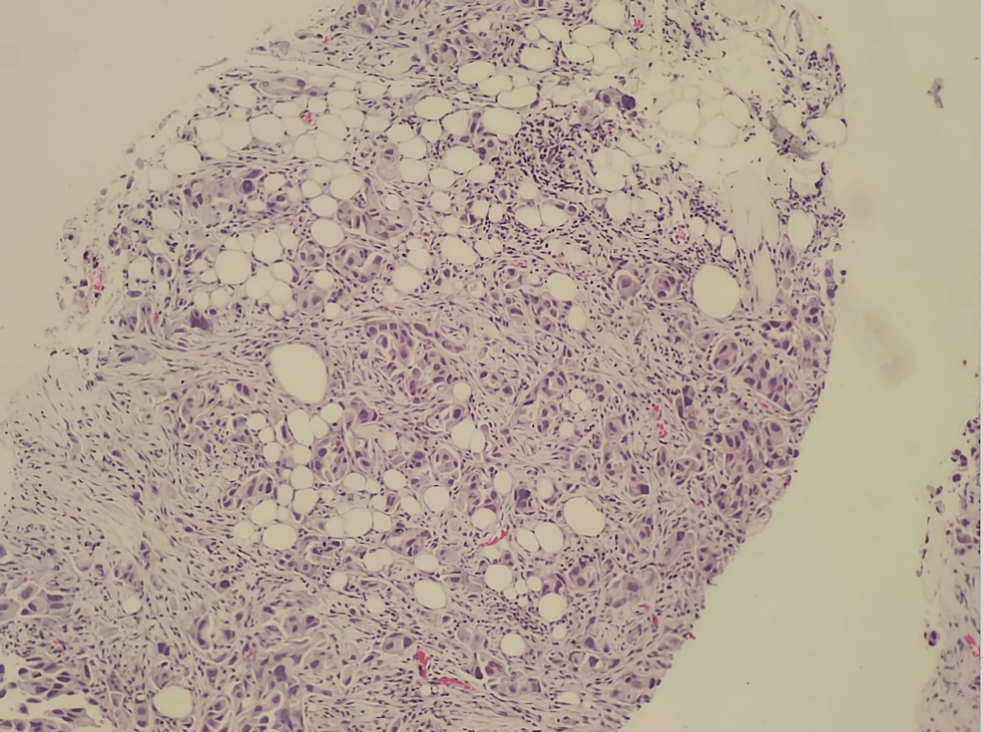
Patient #1: Tru-cut biopsy of the supraclavicular lymph node. Tumor cells are arranged in glands and small nests. Tumor cells are infiltrating into extranodal adipose tissue (H&E stain) (100× magnification). H&E: hematoxylin and eosin

**Figure 2:**
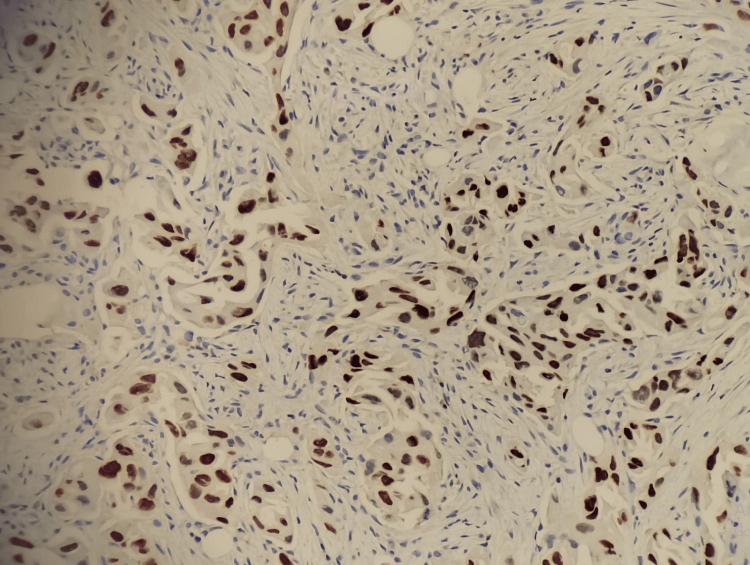
Patient #1: Tumor cells are positive for TTF-1 IHC stain (200× magnification). TTF-1: thyroid transcription factor-1; IHC: immunohistochemistry

The first patient was a 64-year-old Saudi male, ex-smoker, with a history of biopsy-proven lung adenocarcinoma, which was treated with chemoradiotherapy 10 years back. The patient currently presented with a recurrent right lung mass, matted right supraclavicular lymph nodes, and bilateral neck lymphadenopathy, which were suggestive of malignancy. An initial X-ray examination showed a right-sided mediastinal shift. Tru-cut needle biopsy of the right supraclavicular lymph node showed involvement by metastatic moderately differentiated adenocarcinoma arranged in glands and small nests (Figure 1). Tumor cells demonstrated positive expression for cytokeratin (CK) 7, thyroid transcription factor-1 (TTF1), and Napsin A. A thorough immunohistochemical (IHC) examination confirmed primary lung origin after tumor cells revealed positive staining for CK7 and TTF-1 and negative staining for CK20, CDX2, and paired box gene 8 (PAX8) IHC stains (Figure 2).

**Figure 3:**
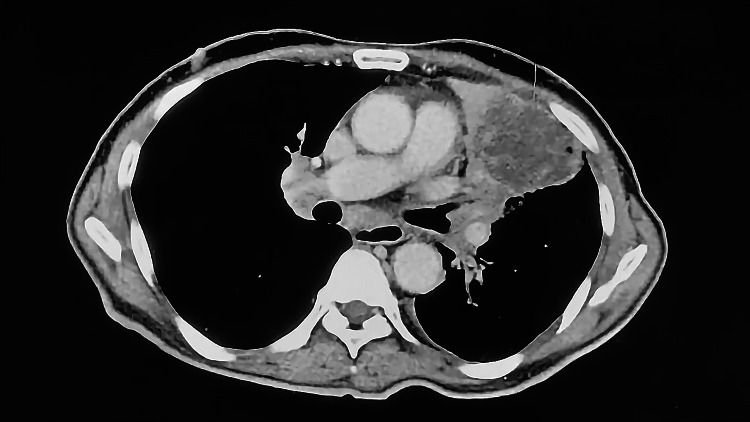
Chest, abdomen, and pelvis CT scan of Patient #1 with contrast (2.9× magnification). CT: computerized tomography

**Figure 4:**
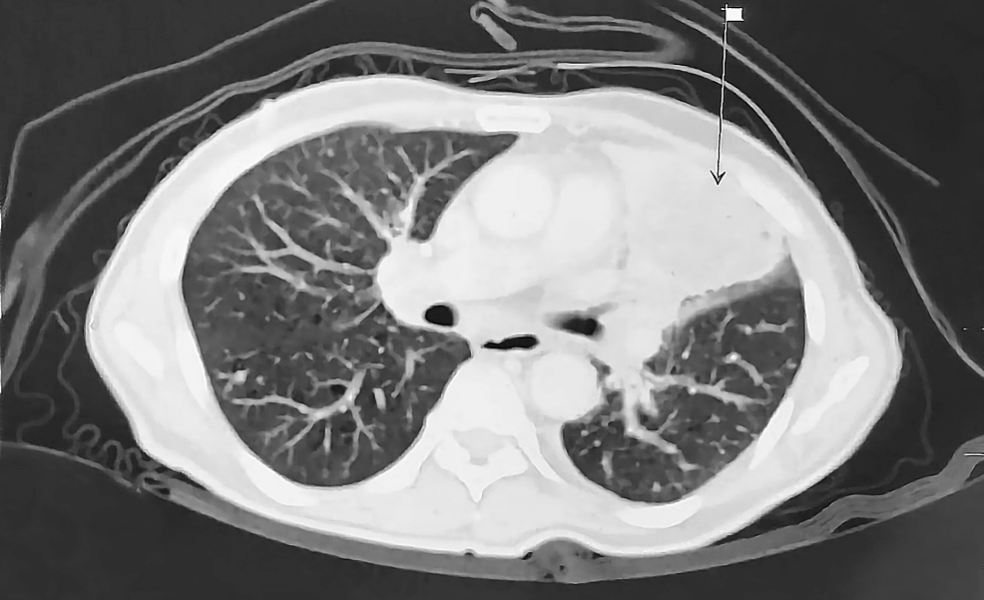
HRCT scan of Patient #1 (2.9× magnification). HRCT: high-resolution computerized tomography

A spiral computerized tomography (CT) of the chest, abdomen, and pelvis region with contrast injection showed thrombosis at the right main pulmonary artery and multiple distant metastatic deposits (Figures 3, 4). On the right side, there was an invasion of the right pulmonary trunk caused by a pathologic process arising in the right lower lobe. Additionally, a large right hilar heterogeneous mass lesion was seen measuring 7 cm × 7.1 cm × 4.2 cm with evidence of mass effect on the surrounding structure with complete encircling of the right main pulmonary truck with thrombus seen inside (positive PE) with its subsequent occlusion of the descending pulmonary artery. Bilateral extensive alveolar infiltrates were observed at the middle lobe, lingual, and basal segments of the upper lung lobes with perivascular/perilymphatic distribution, suggesting early lymphangitis carcinomatosis. Multiple bilateral upper and lower deep cervical lymph nodes were seen, with the largest seen in the right supraclavicular region that became a mass-like lesion measuring 3.7 cm × 1.9 cm with a central hypodense area, suggesting necrosis. Other suspicious cervical lymph node (LN) enlargements were seen at both carotid and posterior triangle spaces, leading to therapeutic doses of SC Clexane.

Case two

**Figure 5:**
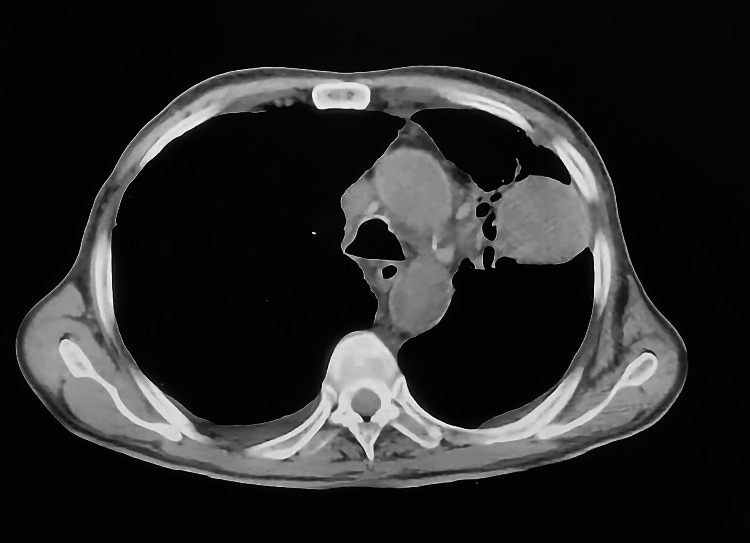
Chest, abdomen, and pelvis CT scan of Patient #2 (2.9× magnification). CT: computerized tomography

**Figure 6:**
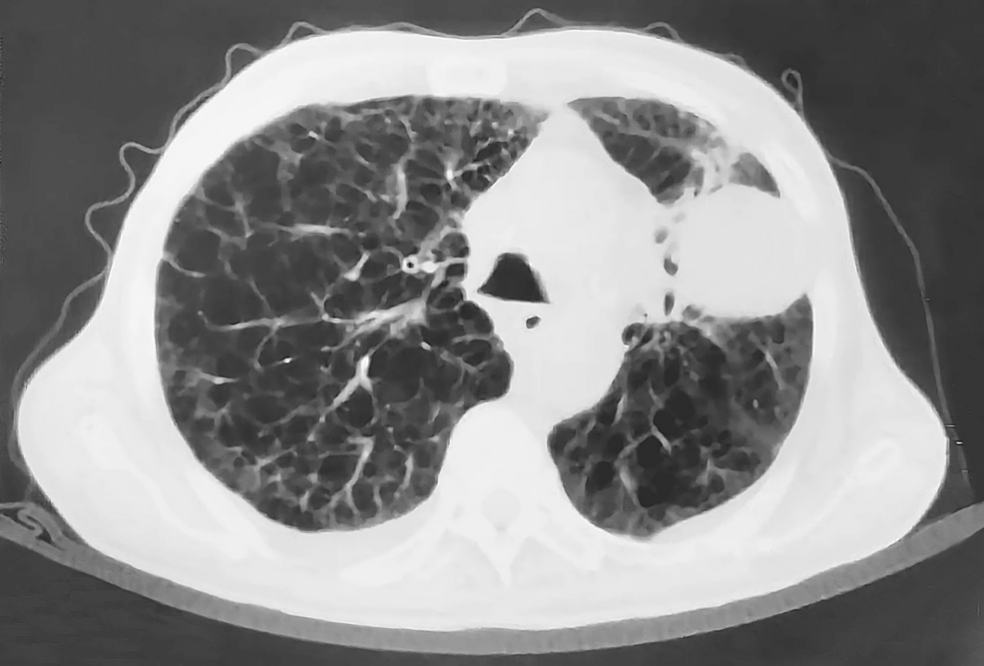
HRCT scan of Patient #2 (2.9× magnification). HRCT: high-resolution computerized tomography

Patient #2 was a 76-year-old Saudi male who presented with biopsy-proven stage III-A left lung adenocarcinoma being treated for respiratory shock. The patient was deemed unfit for surgery due to his old age and unsuitable general physical condition. He was diagnosed with afebrile adenocarcinoma and was vitally stable with fair labs (Neutr 100). Immunostaining tests came up with CK7++, epithelial membrane antigen (EMA)++, TTF1++, CK20, CD15 (Leu M1)++. He received concurrent chemoradiotherapy followed by maintenance chemotherapy. The patient quickly started radiotherapy with chemotherapy was on maintenance PEMETREXED. A later CT of the chest, abdomen, and pelvis scan displayed a remonstration of the peripheral lung pleural-based hypodense mass, which had minimally decreased in size (measuring 4.2 cm × 4.6 cm while previously measuring 4.9 cm × 4.5 cm) (Figures 5, 6). Additionally, the CT chest, abdomen, and pelvis scans showed no distance metastases at the time. However, the patient later developed cores of completely necrotic tumors with viable benign fibro-connective tissue, as observed in the tru-cut biopsy of his aforementioned necrotic cores found in a left lung mass. Following that, he started to develop G-III to G-IV toxicity from the chemotherapy while still sustaining a non-responding left lung mass.

The biopsy material of both patients was subjected to molecular testing for EGFR, KRAS, and MSI genes. No molecular alteration was found in any of the aforementioned targets except for the detection of KRAS G12C mutations in both patients. These mutations were detected using the Idylla™ Real-time PCR KRAS Mutation Test. A summary of the clinicopathological features of the two cases can be found in Table 1.

**Table 1:** Clinicopathological features of the LUAD patients’ KRAS G12C mutation (n = 2). LAUD: Lung adenocarcinoma; KRAS: Kirsten rat sarcoma viral oncogene homolog

Patient	Gender	Age	Diagnosis	Smoking status	Tissue sample
Patient #1	Male	64	Lung adenocarcinoma (non-mucinous)	Ex-smoker	Supraclavicular lymph node biopsy; lung biopsy
Patient #2	Male	76	Lung adenocarcinoma (non-mucinous)	None to mention	

## Discussion

Lung adenocarcinoma is a form of NSCLC which is considered the most common primary lung cancer subtype seen in the United States [17]. Around 52% of lung adenocarcinoma patients have also been reported as having KRAS G12C mutations, making it quite common [18]. Interestingly, according to cancer registry data from various nations in the region, lung cancer has been identified as one of the most frequent cancers in the Middle East and North African regions (MENA) [19,20].

The prevalence of lung cancer in Saudi Arabia contrasts with that of MENA. As of today, Saudi Arabia’s most prevalent cancers are breast, colorectal, prostate, brain, lymphoma, kidney, and thyroid, with prevalence rates of 53%, 50.9%, 42.6%, 9.6%, 9.2%, 4.6%, and 12.9%, respectively. In the context of lung cancer, Saudi Arabia has a low risk of lung cancer. Males had an age-standardized ratio (ASR) of 5.5 per 100,000 in 2013 while females had an ASR of 1.8 per 100,000. In comparison, the average global ASR for males and females in 2008 was 33.8 and 13.5, respectively. Furthermore, Alghamdi et al. reported that out of 404 lung cancer patients from Saudi Arabia who were diagnosed during 2009-2013, NSCLC made up 51.2% of observations (N = 207), of which 105 (50.7%) did not survive, while SCLC made up 48.8% of cases (N = 197), of which 108 (54.8%) did not survive [21]. However, a growing population, a predicted sevenfold increase in the senior population, a high smoking prevalence, which is progressively rising by 1.5% for men and 2.0% for women, and the development of the middle eastern medical field increasing the awareness, diagnosis, and reporting of (e.g., the increased use of imaging like CT and positron emission tomography scans) are all variables that could lead to an increase in lung cancer incidence in Saudi Arabia and the Arabian Gulf more generally, steadily closing the gap between local and international incidence rates [22-25]. This has created a great surge in demand for treatments for this cancer, but the lack of cases concerning KRAS-mutant lung adenocarcinoma in MENA has been a major setback preventing us from learning more about effective treatment plans.

The KRAS gene, which is found on chromosome 12p12.1, is the most frequently mutated oncogene in human malignancies in general, accounting for 22% of all cancers [26]. KRAS4A and KRAS4B are the two major splice variants, having different C-terminal sequences. KRAS4B is the most common kind, but KRAS4A expression is increased in the presence of a tumor [27]. Furthermore, in mice, this has proven to be necessary for the development of lung cancer [28]. KRAS mutations have also been discovered as predictors of poor outcomes in patients undergoing EGFR tyrosine kinase inhibitor (TKI) therapy for EGFR-mutant illness [29]. KRAS has been labeled “undruggable” due to its comparatively smooth protein structure, encouraging scientists to focus on downstream inhibitors to target it. Fortunately, direct-targeting techniques (such as sotorasib) can now be used to limit or prevent KRAS overexpression; however, nothing is known about primary or acquired resistance to such a strategy [30].

KRAS G12C transversion mutations account for 41% of KRAS mutations and are almost exclusively detected in lung adenocarcinoma, with a nearly 90% incidence rate in smokers, which is consistent with our findings wherein both patients who reported their smoking status were smokers [31,32]. When compared to other KRAS mutations, KRAS G12C signaling preferentially activates downstream Ral A/B and RAF/MEK/ERK pathways while decreasing phosphorylated AKT, a factor also seen with KRAS G12V mutations. Because of the mutation’s widespread occurrence, it has been extensively researched, resulting in the development of TKIs such as sotorasib, adagrasib, GDC-6036, JNJ-74699157, and D-1553. Sotorasib, a medicine classified as an irreversible inhibitor with a half-life of roughly six hours, is the most often used TKI for the KRAS G12C mutation [33]. It works by locking KRAS in an inactive, GDP-bound state. Other TKIs are still being explored and tested in clinical trials, whereas adagrasib works similarly [34,35]. Sotorasib and adagrasib are the two most efficacious KRAS inhibitors in NSCLC [36-38].

Ethnicity has been proven to play a big role in influencing the prevalence of KRAS mutations, especially in colorectal cancer and lung adenocarcinoma [39]. For example, multiple studies have confirmed that Asian populations consistently retain a lower prevalence in KRAS-mutant lung adenocarcinoma compared to non-Asians with significant differences, the highest of which, to our knowledge, was a 32.9% vs. 9.3% prevalence rate in non-Asian vs. Asian KRAS mutation rates in lung adenocarcinoma patients [40-43]. Unfortunately, little is known about the prevalence of KRAS mutations in Arab patients, let alone patients in Saudi Arabia or the Arabian Gulf. A study of 106 Lebanese patients with lung adenocarcinoma underwent mutational analysis for KRAS in exon 2 codon 12 and 13 alongside exon 3 codon 61 by reverse hybridization. Of the patients, 37.7% reported KRAS mutations, and 32% exhibited KRAS mutations in codons 12 and 13 of exons 2 and 3 [44]. Moreover, a study on 117 Moroccan lung adenocarcinoma patients aimed to analyze KRAS mutations in codons 12 and 13 of exon 2. Notably, 11/117 (9%) patients reported such mutations, and they were more often observed in males and smokers [45]. These data show a potential discrepancy between Arab populations’ KRAS mutation prevalence. Further studies on large numbers of patients from the Arabian Gulf are needed to confirm patients of the Arabian Gulf compared to the rest of the world in this regard.

The median progression-free survival (PFS), or the time between the start of treatment and the onset of cancer, of various KRAS mutations has also been studied, with a median PFS of 15.57 weeks and overall survival (OS) of 18.64 weeks for KRAS G12C [34,46]. Notably, newer studies have found no link between KRAS mutation status and gender; however, there are strong links between the geographical region and patient age [33]. Furthermore, KRAS G12C NSCLC patients were on average 67 years old and gender distribution was equal. Furthermore, compared to the North/Northeast regions of the world, the South/Southeast sections of the world were reported to be the most commonly afflicted (8.2% and 8.1%, respectively) versus (5.1% and 3.6%, respectively). Finally, patients younger than 50 years had a lower frequency of KRAS G12C mutations (2.0% versus 7.2%, respectively) than those older than 50 years [33].

Our cases not only confirm various claims made by earlier studies regarding KRAS G12C mutations but also present the first documentation highlighting and describing the cases of KRAS G12C-mutant lung adenocarcinoma in patients, which have been possibly missing because of a lack of research. As mentioned before, however, there has yet to be progress regarding inhibitors and drugs that could be used and how they may affect this patient population. We believe that our case report provides a platform for the community to perform large-scale studies on the potential implications of such differences on prognosis.

## Conclusions

Our case study highlights two cases of KRAS-mutant (KRAS G12C) lung adenocarcinoma patients that have never been reported before in the Arabian Gulf. The findings of our study will help clinicians better understand the disease behavior and will open avenues for further molecular research in our region. This will be critical as patients in the Arabian Gulf could witness a sharp increase in lung cancer patients due to factors such as (1) a growing population, (2) a predicted sevenfold increase in the senior population, (3) a high smoking prevalence, which is progressively rising by 1.5% per year for men and 2.0% per year for women, and (4) the development of the middle eastern medical field increasing the awareness, diagnosis, and reporting (e.g., the increased use of imaging such as CT and positron emission tomography scans). Further investigation is needed, however, to identify the most optimal inhibitors and drugs for the patients in the given demographic.
